# Safeguarding Patients, Relatives, and Nurses: A Screening Approach for Detecting 5-FU Residues on Elastomeric Infusion Pumps Using HPLC-DAD

**DOI:** 10.3390/toxics13050416

**Published:** 2025-05-21

**Authors:** Andreia Cardoso, Ângelo Jesus, Luísa Barreiros, Daniel Carvalho, Maria dos Anjos Sá, Susana Carvalho, Patrícia Correia, Fernando Moreira

**Affiliations:** 1 Centro Hospitalar Universitário de São João, Alameda Prof. Hernâni Monteiro, 4200-319 Porto, Portugal; andreiasocardoso1996@gmail.com (A.C.); mar_ga_ri_da20@hotmail.com (S.C.); 2 Escola Superior de Saúde, Instituto Politécnico do Porto, Rua Dr. António Bernardino de Almeida, 4200-072 Porto, Portugal; lsb@ess.ipp.pt; 3 LAQV/REQUIMTE, Escola Superior de Saúde, Instituto Politécnico do Porto, Rua Dr. António Bernardino de Almeida, 4200-072 Porto, Portugal; acj@ess.ipp.pt (Â.J.); pcorreia@ess.ipp.pt (P.C.); 4 LAQV/REQUIMTE, Department of Chemical Sciences, Faculty of Pharmacy, University of Porto, Rua Jorge Viterbo Ferreira 228, 4050-313 Porto, Portugal; 5 i3S—Instituto de Investigação e Inovação em Saúde, Universidade do Porto, 4200-135 Porto, Portugal; danielc@i3s.up.pt; 6 Centro Hospitalar Universitário Do Porto, Hospital de Santo António, 4099-001 Porto, Portugal; mariadosanjosrochasa@gmail.com

**Keywords:** cytotoxic, chemotherapy, antineoplastic, 5-FU, HPLC-DAD, occupational exposure, colorectal cancer

## Abstract

**Background/Objectives:** The leakage of 5-fluorouracil (5-FU) from elastomeric infusion pumps used in cancer therapy poses a potential risk of unintentional exposure to multiple individuals, including patients’ relatives and healthcare professionals, and may also compromise the accurate administration of 5-FU dosages to patients. This study aimed to develop, validate, and apply an analytical method to detect and quantify 5-FU residues on the external surfaces of infusion pumps. **Methods:** A high-performance liquid chromatography with diode-array detection (HPLC-DAD) method was optimized for the quantification of 5-FU contamination across different components of the infusion pump, including the hard casing, infusion tubing, and catheter connection port. A mobile phase containing 5% acetic acid was used to obtain more efficient separation of 5-FU and the detection was performed at 260 nm. The method was evaluated for linearity, sensitivity, precision, accuracy, selectivity, robustness, and stability. **Results:** The method demonstrated linearity within the range of 0.150 to 3.000 µg/cm^2^, with limits of detection and quantification of 0.05 µg/cm^2^ and 0.14 µg/cm^2^, respectively. Relative standard deviations ranged from 1.8% to 12.7%, and accuracy exceeded 85%. In real sample analysis, detectable residues were found around the catheter connection port. **Conclusions:** This screening-oriented method addresses an existing gap, as previous contamination reports were based solely on self-reported user observations. The detection of 5-FU residues highlights the critical need for safe handling practices and the consistent use of personal protective equipment (PPE) to protect healthcare workers, especially nursing staff involved in the removal of the infusion pumps, after treatment.

## 1. Introduction

Cancer is currently one of the two main causes of premature death worldwide, along with cardiovascular diseases. Projections suggest that over the course of the 21st century, cancer will become the leading cause of death in most countries [1]. In terms of current incidence and future projections, the scope of cancer is equally impressive: approximately 20 million new cases were diagnosed in 2022, and projections point to 35 million new cases by 2050 [2]. One of the most frequently diagnosed cancers in 2022 was colorectal cancer (CRC) (9.6% of cases), only surpassed by lung cancer (12.4% of cases) and female breast cancer (11.6% of cases). In terms of mortality, CRC was the cause of almost 1 million deaths worldwide in 2022. It was the second deadliest cancer (9.3% of deaths), only surpassed by lung cancer (18.7% of deaths) [2]. Despite the high incidence and mortality of CRC in Western countries, over the last few decades, the prognosis of CRC patients has, slowly but steadily, improved. Early diagnosis, the expansion of ablative techniques and the more strategic administration of systemic chemotherapy have all contributed to this improvement [3]. The regimen commonly referred to as “FOLFOX” (composed 5-FU, leucovorin, and oxaliplatin) is the standard chemotherapy treatment for CRC [4,5]. In this scheme, 5-FU is often administered by bolus in the hospital unit, followed by administration of the same drug at home using a medical device called an infusion pump, which allows continuous, slow infusion for 46 h. Although there might be differences based on the patients’ clinical and biological characteristics, it can be said that 5-FU is very likely to be administered using infusion pumps to patients with CRC [6]. 5-FU is an antimetabolite drug that has been widely used since 1957 to treat different types of cancer, such as gastric adenocarcinoma, pancreatic adenocarcinoma, breast carcinoma, and, for the reasons described above, colorectal adenocarcinoma. The primary mechanism of action of 5-FU involves both following effects: the inhibition of thymidylate synthase, an enzyme crucial for deoxythymidine monophosphate (dTMP) synthesis required for deoxyribonucleic acid (DNA) replication and repair, and the misincorporation of 5-FU as a pyrimidine analog into ribonucleic acid (RNA) and DNA in place of uracil or thymine [7]. Initially, the administration of 5-FU was restricted to intravenous bolus injection. However, bolus administration is very limited by the extremely short half-life of 5-FU. Since the mid-1970s, 5-FU infusion regimens have gained increasing acceptance in clinical practice, due to their improved efficacy and safety profile compared to intravenous bolus regimens. The administration of 5-FU intravenously over 46 h has since become the standard protocol for administering this drug and is used in combination chemotherapy regimens for CRC [8]. The administration of chemotherapy on an outpatient basis using portable, disposable infusion pumps has represented a major advance in clinical oncology. This treatment modality makes it possible to carry out cancer treatment for a short period (30 min to 12 h) or longer (1 to 11 days), in the comfort of the patient’s home and without the assistance of a healthcare professional, while also relieving the pressure on the material and human resources of healthcare facilities [9,10,11]. Infusion pumps come in various types, which can be classified as volumetric, syringe, elastomeric, or electric. Volumetric pumps are used for the administration of drugs and nutrition (enteral and parenteral). Syringe pumps are employed for self-controlled pain management, enteral and parenteral nutrition, blood and blood products in neonatology and pediatrics, and insulin delivery. Elastomeric and electric infusion pumps are exclusively used for drug administration, with electric pumps using a battery-powered fluid pumping mechanism connected to a drug solution reservoir [12]. Currently, infusion pumps are widely used in hospitals and home care settings to administer chemotherapy, antimicrobials, analgesics, and anesthetics, as well as for postoperative pain control and chronic pain management [13]. Infusion pumps can be primarily classified based on the following attributes (when applicable): operating mechanism (mechanical or electronic), pumping mechanism (elastomeric, spring, or vacuum), drugs pumped (chemotherapy, insulin, analgesics), administration setting (hospital or ambulatory), and safety features (alarm or software-based) [11]. Elastomeric infusers are frequently employed due to their ease of use, portability and fewer technical problems, resulting in alarms. In elastomeric pumps, fluid pressure is generated by the force of one or more stretched elastomers that can be natural or synthetic, such as isoprene rubber, latex, and silicone. The external protector can also be made of a soft elastomer, providing a smaller disposal space, or a harder plastic that offers greater protection against sharp objects, commonly referred to as a hard shell [13].

Notwithstanding the clinical advantages of cytotoxics in the treatment of cancer, these medications are known for the intensity and severity of their adverse reactions. In addition to cancer patients, various healthcare workers, notably nurses (responsible for administration) and pharmacy technicians and pharmacists (responsible for preparation), are exposed to these carcinogenic, mutagenic, and reproductively harmful medications. It is imperative to use the most appropriate equipment and materials and to define safe handling and administration practices among health professionals, since a higher incidence of DNA damage, chromosomal abnormalities, and cancer has already been revealed among occupationally exposed individuals compared to control groups [14,15,16]. A meta-analysis concluded that, in 62.5% of the studies considered (*n* = 24), the frequency of micronuclei (a biomarker for measuring chromosome damage and loss) was higher in healthcare workers occupationally exposed to cytotoxic drugs when compared to control groups [17].

In the specific case of 5-FU administered at home via infusion pumps, the possibility of patients’ relatives and even pets being inadvertently exposed to the drug cannot be ruled out. 5-FU is a recognized cause of toxicosis in dogs, and the death of animals has already been described a few hours after ingestion [18]. A recent retrospective study found that leaks of cytotoxic drugs from infusion pumps were the main incident reported in materiovigilance notifications, contributing to 44% of the 205 cases reported between 2017 and 2021 in a Moroccan hospital unit [9].

The social, demographic, and even economic impact of cancer is massive [1,19]. It is therefore essential that health policies, the education of health professionals, and the development of drugs and devices seek to maximize the effectiveness of treatment, but also to promote the safety of patients’ families and professionals. Accordingly, this study aims to (i) develop and validate an analytical method using HPLC-DAD for the detection of 5-FU on infusion pumps’ external surfaces; and (ii) to assess the potential deposition of 5-FU residues on the outer faces of infusion pumps in a real-life context.

## 2. Materials and Methods

Methanol (≥99.8%; 32.04 g/mol), acetonitrile (≥99.9%; 41.05 g/mol), and 5-FU standard (99%; 130.08 g/mol) were purchased from Fisher Scientific (Loughborough, UK). Acetic acid (60.05 g/mol) was purchased from VWR^®^ (Fontenay-sous-Bois, France). Ultrapure water was produced using a Barnstea^®^ Smart2Pure^®^ Water Purification System (Thermo Scientific^®^, Waltham, MA, USA). Ethyl acetate (99.8%; 88.10 g/mol) was acquired from AppliChem Panreac (Darmstadt, Germany). Lastly, cyclophosphamide (>98% purity, 279.10 g/mol) was obtained from Sigma-Aldrich (St. Louis, MO, USA).

Chromatographic analysis was performed using a JASCO^®^ (Oklahoma City, OK, USA) LC-4000 series HPLC system equipped with a diode array detector (DAD). A reversed-phase C18 column (Hypersil-GOLD, 150 × 4.6 mm, 5 µm; Fisher Scientific, Loughborough, UK) was used as the stationary phase. ChromNav^®^ 2.0 software was used for data acquisition and analysis.

Different compositions of the mobile phase were tested during the analytical method development, all operating isocratically. The mobile phase initially tested consisted of acetonitrile, methanol, and purified water, in a ratio of 19:13:68 (*v*/*v*/*v*), as previously described by Viegas et al. [20], but the finally selected mobile phase composition consisted of 5% (*v*/*v*) acetic acid in purified water. Purified water was previously filtered through a 0.45 µm acetate-cellulose filter (Sigma-Aldrich (St. Louis, MO, USA)). All mobile phase components were degassed by ultrasounds (Bandelin Sonorex^®^, Sigma-Aldrich (St. Louis, MO, USA)) at room temperature (±25 °C) for 30 min. An injection volume of 10 µL, a flow rate of 0.800 mL/min, and a run time of 10 min were also applied. The possibility of simultaneously monitoring different wavelengths during a single chromatographic run, characteristic of the DAD methodology, was exploited in this study to evaluate the intensity of 5-FU detection under different wavelengths reported in the literature: 254 nm [21,22]; 260 nm [23,24]; 265 nm [25,26]. After analysis, 260 nm was the chosen wavelength.

To prepare the 5-FU stock solution, the standard was dissolved in a solution of acetonitrile, methanol, and purified water (19:13:68, *v*/*v*/*v*) with vortex stirring (VWR^®^ VV3) for 3 min at maximum speed. The resulting 5-FU 1 mg/mL stock solution was stored under refrigerated conditions (4 °C).

To ensure that the method performance was assessed under conditions mimicking the analysis of real 5-FU residues on infusion pumps, validation was performed using gauze dressings deliberately spiked with defined amounts of 5-FU. Therefore, all sensitivity and analytical performance parameters reflect the method’s effectiveness for detecting 5-FU in the actual gauze matrix (preliminary analyses revealed no interferences or changes in chromatographic profiles between extracts from gauze samples and standard solutions). Under laminar flow conditions, all gauze dressings used during the method validation (non-woven 10 cm^2^ gauzes from ADA, Porto, Portugal) were moistened with ethyl acetate and spiked with 5-FU (except blanks). Deliberate contamination with a standard 5-FU solution resulted in gauze dressings with concentrations of 0.150 µg/cm^2^, 0.375 µg/cm^2^, 0.750 µg/cm^2^, 1.125 µg/cm^2^, 1.500 µg/cm^2^, 2.250 µg/cm^2^, and 3.000 µg/cm^2^. Blank dressings were also prepared without 5-FU spiking. Moistening with ethyl acetate simulated real conditions and allowed the assessment of potential matrix effects from ethyl acetate in the chromatographic analysis. For real sample analysis, no spiking was performed, and samples were collected by wiping real infusion pumps. The matrices used for real sample analysis were the same as those used for validation: non-woven 10 cm^2^ gauzes from ADA moistened with ethyl acetate. The extraction procedure was then performed.

The extraction method was adopted from a procedure previously described for the extraction of 5-FU after wipe sampling to assess surface contamination [20,27]. For that purpose, the gauze dressings were added to 15 mL of acetonitrile/methanol/water (10:25:65, *v*/*v*/*v*), and the extraction was carried out by vortex stirring for 1 min at maximum speed. The obtained extract was then filtered using a 0.20 µm polytetrafluoroethylene (PTFE) filter (Chromafil^®^ Xtra PA, Sigma-Aldrich (St. Louis, MO, USA)), directly into amber glass vials for immediate chromatographic analysis.

To assess linearity, the calibration curve was constructed using gauze dressings spiked with different amounts of the 5-FU stock solution, resulting in seven known concentrations of 5-FU (0.150 µg/cm^2^, 0.375 µg/cm^2^, 0.750 µg/cm^2^, 1.125 µg/cm^2^, 1.500 µg/cm^2^, 2.250 µg/cm^2,^ and 3.000 µg/cm^2^), which were subjected to the previously described extraction procedure. All the calibration standards were analyzed in triplicate, as were the blanks (sample matrix without 5-FU). A total of nine independent calibration curves were performed, on seven different days. A calibration line comprising all analyses was constructed. The relative standard deviation (RSD) of the slope is one method for assessing linearity [28,29]. Accordingly, Equation (1) was used to calculate RSD:

RSD (%) = *s_m_*/*m* × 100, (1)

where *m* stands for the slope and *s_m_* for the slope standard deviation.

Following the recommendations, the limit of detection (LOD) and limit of quantification (LOQ) were calculated using the calibration curve slope, *m*, and the standard deviation *δ* of the response obtained for the analysis of 12 blank samples analyzed in triplicate [28,30], according to Equations (2) and (3):

LOD = 3.3 × *δ*/*m*, (2)

LOQ = 10 × *δ*/*m*, (3)

For evaluating repeatability (also known as intra-day precision), the concentrations of 0.375 µg/cm^2^, 1.125 µg/cm^2,^ and 2.250 µg/cm^2^ of 5-FU were selected, and the peak areas of the respective chromatograms were obtained by HPLC-DAD analysis.

The same concentrations of 5-FU were evaluated for intermediate precision (also known as inter-day precision) (0.375 µg/cm^2^, 1.125 µg/cm^2^, and 2.250 µg/cm^2^ of 5-FU), but on three different days. Analysis on different days is a commonly introduced variable to assess intermediate precision [28,31].

Both repeatability and intermediate precision were evaluated using artificially prepared samples (after enrichment with relevant amounts of the analyte) and expressed under RSD [28]. The RSD considered the standard deviation of the peak areas of the chromatograms of the extracted samples, *s*, and the respective averages, *X* (Equation (4)):

RSD (%) = *s*/*X* × 100 (4)

where s represents the standard deviation of a series of measurements, and X represents the mean value of the independent variable.

To assess the method’s accuracy, a spiking study was performed. This is one of the strategies recommended by the International Council for Harmonisation of Technical Requirements for Pharmaceuticals for Human Use (ICH) guidelines [28] to assess accuracy. An appropriate number of determinations and concentration levels covering the reportable range were used, namely three concentrations/three replicates each: 0.375 µg/cm^2^, 1.125 µg/cm^2^, and 2.250 µg/cm^2^. Accuracy was expressed as the mean percent recovery of the known analyte added to the sample. To perform the spiking study, different volumes of the 5-FU stock solution were spiked in gauze dressings, resulting in the above-mentioned concentrations (C_t_). After extraction and analysis, the concentration was back-calculated using the peak areas and the calibration equation. Then, the experimentally obtained results (C_e_) were compared with nominal/theoretical concentrations (C_t_) using Equation (5):

Accuracy = (C_e_/C_t_) × 100 (5)

Three gauze dressings were spiked with both 5-FU and cyclophosphamide to assess specificity. The spiking resulted in a final concentration of 1.125 µg/cm^2^, of both compounds. All three gauze dressings were analyzed in triplicate.

Robustness was evaluated based on minor changes in the mobile phase’s pH and flow rate, as per ICH guidelines [28], including a 0.2 decrease in pH and a 0.2 mL/min increase in flow rate, with resulting differences in peak area and retention time calculation as compared to the optimized method. The evaluation of robustness was performed with gauze dressings spiked with 1.125 µg/cm^2^ of 5-FU, in triplicate.

To analyze the stability of 5-FU, different concentrations of spiked samples were analyzed (0.150 µg/cm^2^, 0.750 µg/cm^2^ and 3.000 µg/cm^2^) which were stored at room temperature (±25 °C), refrigerated (4 °C) and frozen (−20 °C), and analyzed again after three and five days. Stability (%) was then calculated using Equation (6), using the average area of the peaks analyzed for each concentration at the start of the stability study (Ā_0_) and the average area of the peaks analyzed for each concentration at the end of the stability study (Ā_i_), where i stands for 3 (after 3 d) or 5 (after 5 d).

Stability _i_ = (Ā_i_)/(Ā_0_) × 100 (6)

For the analytical evaluation of the presence of 5-FU residues on the outside of infusion pumps used in chemotherapy in a real-life context, a request for authorization to carry out this study was submitted to the Department of Education, Training and Research of the Centro Hospitalar Universitário do Porto (CHUP) and its Ethics Committee. The request was approved under the number 2022.112 (086-DEFI/088-CE). On two different days, gauze dressings moistened with ethyl acetate were used to wipe the external parts of ten infusion pumps that had been used for oncology treatments. The sampling occurred at the exact moment of the removal of the infusion pumps by the nursing staff when the administration of 5-FU had supposedly already ended. The study focused on sampling only those elastomeric pumps specifically utilized within the hospital where the research was conducted. As previously described for validation purposes, 10 cm^2^ gauze dressings were used. The samples were taken from three different areas of the infusion pumps: the outside of the pump (hard casing), the infusion wire (an integral part of the infusion pump), and the catheter connection port. After wiping, the gauze dressings were placed in sterile Petri dishes, sealed and immediately transported to the laboratory for analysis, in a journey lasting less than 20 min. The extraction and HPLC-DAD analysis procedures were carried out following the conditions and procedures described in Sections 4.2 and 4.4, respectively. Accordingly, the gauze dressings were added to 15 mL of acetonitrile/methanol/water (10:25:65, *v/v/v*), vortex stirred for 1 min at maximum speed, and the extract was then filtered through a 0.22 µm PTFE filter. As performed during the validation, 5% (*v/v*) acetic acid was used as mobile phase for chromatographic separation and 260 nm was the wavelength used for detection. The identification of 5-FU in the samples was performed by comparing the retention time with that of a high-purity reference standard.

## 3. Results

### 3.1. Method Development and Optimization

The analytical method was initially based on the conditions previously described by Viegas et al. [20], with a mobile phase consisting of acetonitrile, methanol, and purified water, in a ratio of 19:13:68 (*v/v/v*). After observing the overlap of the rapidly eluting 5-FU chromatographic peak with the injection peak, because of pressure variations at the time of injection, other mobile phase conditions were tested. Given the better peak resolution with increased proportions of purified water and enhanced acidification with acetic acid, sustained increases of water volume (75, 85, 95, and 100%) were evaluated. At the same time, increments of 0.5% acetic acid volume were tested, between 0.5 and 5% (*v/v*).

**Figure 1:**
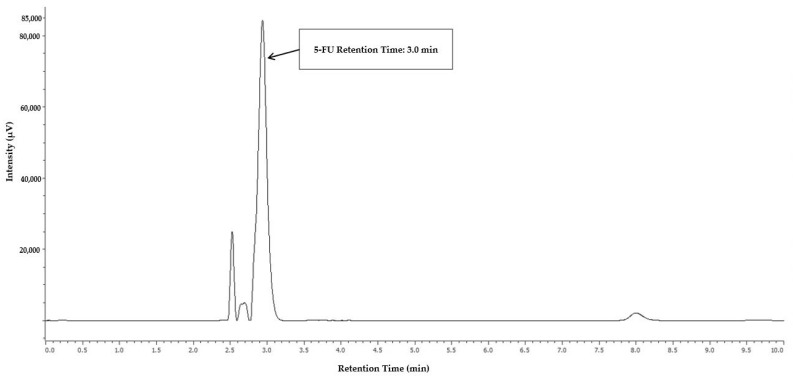
Chromatogram of 5-FU calibrator, exhibiting the retention time at 3.0 min after extraction of a gauze dressing spiked with 3.000 µg/cm^2^ of 5-FU.

Among the various conditions evaluated for 5-FU separation and detection, the mobile phase composed of 5% (*v/v*) aqueous acetic acid resulted in the best chromatographic profile and separation of the analyte peak from the injection peak, and the detection at 260 nm allowed a higher signal intensity. Figure 1 displays the chromatographic profile of 5-fluorouracil (5-FU), with a retention time of 3.0 min.

### 3.2. Method Validation

#### 3.2.1. Linearity

The final calibration curve was the result of integrating the nine independent curves and showed a quadratic correlation coefficient (R^2^) of 0.9987. The obtained equation is expressed in Equation (7).

Area = 212,110 × Concentration + 83,304(7)

To further assess linearity, the chromatographic peak areas used to develop the linear method were also back-calculated to evaluate their accordance with the 5-FU theoretical concentrations (Table 1).

#### 3.2.2. Detection and Quantification Limits

After analyzing the replicate blanks (*n* = 36), and using the slope value of the calibration curve line, it was possible to calculate the LOD and LOQ of the method, which resulted in values of 0.05 µg/cm^2^ and 0.14 µg/cm^2^, respectively.

#### 3.2.3. Precision

Table 2 shows the intra- and interday precision results, with repeatability RSD values at or below 3.2% and intermediate precision RSD values at or below 12.7%.

#### 3.2.4. Accuracy

Table 3 depicts the accuracy of the method, obtained after performing the spiking study.

#### 3.2.5. Specificity/Selectivity

To assess the specificity of the method, a solution of cyclophosphamide alongside the solution containing 5-FU was used to spike gauze dressings. Cyclophosphamide exhibited a retention time of 8.2 min, with its peak distinctly identifiable from 5-FU for accurate quantification.

#### 3.2.6. Robustness

Robustness was evaluated by minor adjustments in the mobile phase’s pH and flow rate. By adjusting the mobile phase’s pH (−0.2), both 5-FU peak area and retention time values maintained the same profile as the optimized method. As to the adjustment of the flow rate (+0.2 mL/min), a reduction in the 5-FU peak area and retention time was observed (Table 4).

#### 3.2.7. Stability

The results of stability studies of 5-FU after 3 and 5 d of storage under different conditions (−20 °C, 4 °C, and room temperature) are depicted in Table 5.

### 3.3. Analysis of Real Samples

**Figure 2:**
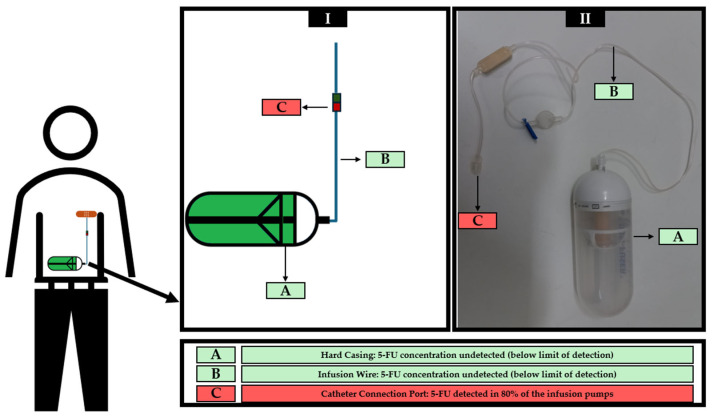
Schematic representation of the placement of infusion pump during administration and identification of 5-FU sampling site in a drawn infusion pump (**I**) and a real infusion pump (**II**).

Ten infusion pumps actually used in cancer treatments were wiped sampled in three different regions, according to Figure 2, generating a total of 30 samples, which were analyzed in triplicate. The results obtained after HPLC-DAD analysis are shown in Table 6.

**Figure 3:**
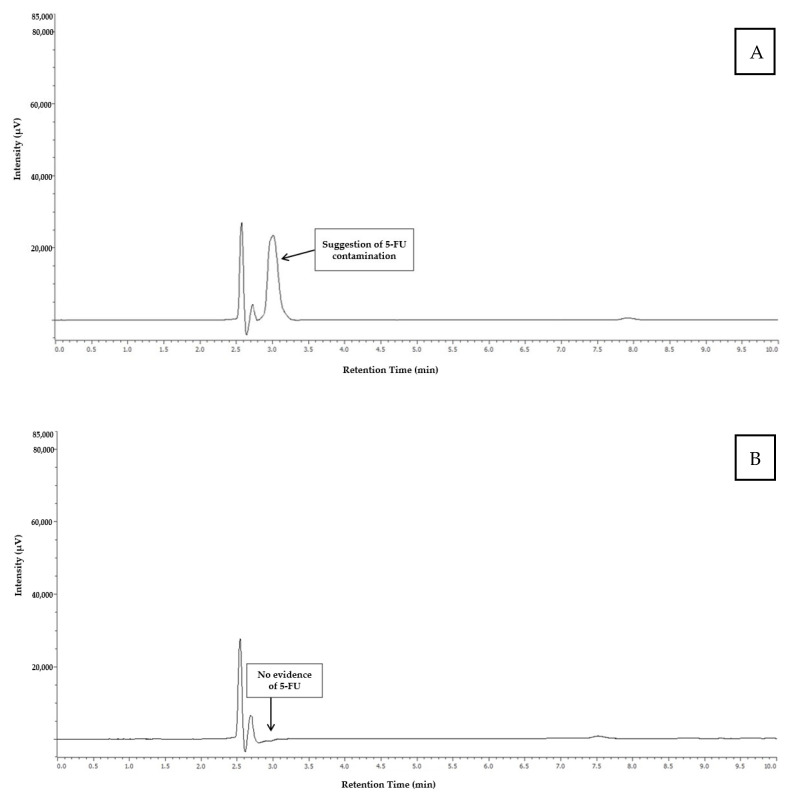
Chromatogram obtained from the analysis of the sample collected at the catheter connection site of infusion pump 4 (**A**) and the infusion wire of infusion pump 5 (**B**).

Figure 3 shows the chromatograms obtained from the analysis of the catheter connection port of the infusion pump 4 (indicating the presence of 5-FU), and from the infusion wire of the infusion pump 5 (indicating the absence of 5-FU).

## 4. Discussion

Chromatographic methods, such as HPLC-DAD and gas chromatography coupled with mass spectrometry (GC-MS), are essential tools for screening in various situations that pose risks to human health, including doping, poisoning, and intoxication. Their features allow for the simultaneous detection of multiple substances in different matrices, enabling rapid and accurate diagnostics in both clinical and forensic settings [32].

Among analytical techniques, HPLC-DAD stands out not for its exceptional sensitivity or specificity, but for its practicality, accessibility, and broad implementation across laboratories worldwide. Unlike more advanced and costly platforms such as LC-MS/MS, which are ideal for confirmatory analyses, HPLC-DAD offers a convenient and sufficiently robust solution for initial screening purposes. The widespread availability of this method in academic, hospital, and industrial settings makes it an attractive option for monitoring potential contamination, substance leakage, or exposure risks in a variety of contexts. Moreover, the dissemination of validated HPLC-DAD methodologies within the scientific community encourages broader adoption, particularly in institutions or regions where access to mass spectrometry is limited. Although HPLC-DAD lacks the molecular confirmation capabilities of more sophisticated detectors, its cost-effectiveness, simplicity, and adequacy for preliminary detection make it a valuable tool in situations requiring timely and practical screening, including quality control, environmental monitoring, and exposure assessment in healthcare environments [33]. Thus, chromatographic methods are indispensable screening tools in scenarios demanding rapid and reliable results, contributing significantly to public health and individual safety.

The choice of chromatographic conditions that promoted a more symmetrical chromatographic profile and more efficient separation concerning the injection peak was relevant as it allowed for more accurate quantification of the analyte. Given that 5-FU is an extremely polar compound, with a n-Octanol/Water Partition Coefficient (log K_ow_) of −1, reversed-phase chromatographic analysis is challenging due to the rapid elution of the compound [34]. The reduction or elimination of organic solvents in the mobile phase, with the consequent increase in the aqueous phase, permits the increase in the retention time of polar compounds, which justifies the observed results. 5-FU is a weak acid with an acid dissociation constant (pKa) of around 8.0 [35], thus, acidification of the mobile phase increased the portion of the total molecules present in the non-ionized form, leading to higher retention times [36]. Therefore, the use of acetic acid in the mobile phase allowed the pH adjustment to better separate the compounds [37] and it is not very aggressive to the equipment at the used concentrations (5% *v/v*). Acetic acid is also recognized as one of the most commonly used acids in chromatography alongside phosphoric acid and formic acid [38]. But the use of this particular weak organic acid confers an environmental improvement and can be considered safe as this reagent is commonly found in food like vinegar [39] and even in nature, being frequently produced by several bacteria [40]. Additionally, it is not adsorbed on particles or sediments and is biodegradable, although it is slightly toxic to most microorganisms at concentrations as low as 0.5% (*v/v*) [38]. Previous studies have already described the use of acidified aqueous mobile phases without organic solvent to detect 5-FU in reversed-phase chromatography [41,42]. The method herein described has the advantage of presenting the use of a more innocuous acid than perchloric acid [41] and orthophosphoric acid [42], thus depicting a new, safe, and greener HPLC method for this purpose. The higher signal intensity provided by detection at a wavelength of 260 nm turned out to be important because it provided greater sensitivity for the analytical method. This difference compared to the other tested wavelengths can make a difference when dealing with the detection of trace quantities.

The performance of the analytical method was assessed according to ICH guidelines and encompassed the following parameters: linearity, detection and quantification limits, precision, accuracy, specificity/selectivity, robustness, and stability [28]. In the context of the evaluation of analytical methods for multiple different analytes such as medicines, drugs of abuse, contaminants, and bioactive compounds, it is usual to determine the performance of the method towards all, or at least some, of the analytical parameters herein described and assessed [43,44,45,46,47].

Nine independent calibration curves comprising seven concentration levels (all of them analyzed in triplicate, for each independent calibration curve) were combined to generate a final calibration curve for the evaluation of linearity. Accordingly, mandatory requirements of a minimum of five concentration levels were conveniently addressed [28,48]. Linear regression was evident between the peak area and analyte concentrations, ranging from 0.150 to 3.000 µg/cm^2^ with a correlation coefficient greater than 0.999. Due to its closeness to 1, this value is a strong evidence of the method’s linearity [31,49,50]. Notwithstanding, it is recognized that a non-linear relationship can give rise to correlation coefficient values close to 1. It is therefore advisable to evaluate the deviation of the actual data points from the regression line. The difference between the real values used to construct the calibration curve and the back-calculated values using the equation generated in the model ranged from 97% to 104%. The deviation must show random behavior in a constant range, without systematic patterns or regularities, which turned out to be the case [28,51].

The results obtained for the calculation of LOD (the lowest concentration of a substance in a sample that can be consistently detected) and LOQ (the lowest concentration of a substance in a sample that can be consistently quantified) show that they are within the same order of magnitude as previous studies using identical HPLC-DAD technology, after extraction of 5-FU from gauze dressings [20,27]. The LOD and LOQ values obtained in the present study (0.05 µg/cm^2^ and 0.14 µg/cm^2^, respectively) are slightly higher than those reported by Viegas et al. [20,27] (LOD of 0.01 µg/cm^2^ and LOQ of 0.033 µg/cm^2^), but it is important to note that, in those studies, the injection volume was 100 µL, whereas in the present study, the injection volume was 10 µL, i.e., 10 times lower. The injection of higher volumes necessarily results in a higher sensitivity and lower limits for the quantification and detection of the analyte. However, the use of reduced volumes is of analytical and environmental interest, as it allows for a higher number of analyses for each standard or sample and generates less waste. Table 7 presents studies in which 5-FU was detected in the same matrix as in the present study (gauze dressings). As expected, the LOD and LOQ obtained in our study are higher than those reported in studies using LC-MS/MS, given the inherently greater sensitivity of LC-MS/MS compared to HPLC-DAD.

As recommended, nine determinations covering the reportable range for the procedure (three concentrations/three replicates each) were assessed to evaluate precision parameters (both intra-day precision/repeatability and intermediate precision) [28]. The method herein depicted demonstrated that RSD values remained below 15% for each analyzed standard concentration, confirming the method’s repeatability and intermediate precision, despite minor variations in analytical conditions [49,57].

Following ICH recommendations, accuracy was established across the tested range of the analytical procedure under its regular test conditions (namely the presence of matrix) and demonstrated by comparing the experimentally obtained values with expected ones [28]. Therefore, three samples of gauze dressings were fortified with concentrations of 5-FU present in the linearity range, specifically for the evaluation of this parameter. The samples were extracted and analyzed following the normal procedure. The values obtained were then back-calculated to compare them with the expected theoretical value, to assess the deviation from the linear model, as described in other studies [58,59,60]. The accuracy of the method, as indicated by the recovery values of standard 5-FU within an acceptable range of 85.1–99.7%, shows its suitability for the quantification of this compound. A relative error of no more than 15% is suggestive of adequate accuracy of the analytical method [49].

The specificity/selectivity can be demonstrated by evidencing “absence of interference”. This consists of proving that the detection or measurement of an analyte remains unaffected by other substances, namely other components likely to be present [28]. Given that the preparation of the infusion pumps must be carried out in laminar flow chambers where other cytotoxic drugs are usually handled [61], the external surface of the infusion pumps could become contaminated. Hence, the possible modification of the chromatographic signal was evaluated in the presence of one of the most commonly cytotoxic handled drugs: cyclophosphamide [51,62]. The method exhibited good selectivity, clearly separating both compounds with high resolution.

The ICH guidelines stipulate that an HPLC method’s robustness might be evaluated by introducing modifications in the mobile phase and testing the method’s performance in these conditions [28]. In this study, changing the pH maintained practically the same intensity of the 5-FU chromatographic peak obtained with the optimized method, without affecting retention time. Since high concentrations of acid in the mobile phase may harm the HPLC system, it was concluded that the adopted method was more adequate [63]. Nevertheless, regarding this particular modification, it was concluded that the method was robust. As to the modification in the flow rate, the faster elution could result in a more efficient method. However, there was a significant decrease in the chromatographic peak area of 5-FU, by an order of 30%. This means that if the equipment is not properly calibrated in terms of flow rate, the results can be significantly affected in terms of 5-FU analysis, compromising its quantification.

The stability of an analyte in solution at different storage temperatures (room temperature, refrigeration, and freezing) must be assessed at three concentrations and in triplicate, meeting the mandatory requirements [48]. Concerning the stability of the 5-FU solutions, and from a quantitative point of view, it was possible to see that, in most cases, there is a decrease in stability over time. Stability values above 100% can be explained by the evaporation or loss of the extraction solvent, causing an increase in the concentration of 5-FU in the sample. It is assumed that, for a compound to be considered stable, there must be no variation of more than 15% in comparison to the values obtained in the initial standard solution (t0) [49]. In other words, the stability values must be between 85 and 115%. Most of the results considered “unstable” were observed after 5 d of storage. This may mean that, especially for quantification purposes, the results obtained from the study described here may be inaccurate in the case of infusion pumps used for more than 3 d. Storage at room temperature showed the most stable results for all concentrations and storage days. A potential justification is the precipitation of 5-FU at low temperatures [64].

Previous research reported the occurrence of 5-FU leaks and spills from infusion pumps used in patients’ homes [9]. However, monitoring the performance of infusion pumps cannot be limited to incidents reported by patients. Trace amounts of 5-FU resulting from spillages may not be visible to the naked eye. In addition, accurate estimation of spillages is important to assess potential impact on treatment success. According to the authors’ knowledge, this is the first study proposing an analytical method specifically for assessing the safety of infusion pumps regarding spillages of 5-FU. User reports of leaks and existing research highlighting the poor performance of various medical devices in clinical settings [65,66], despite being readily available, underscore the critical need for material surveillance in this area. The development and validation of analytical methods to assess the suitability and performance of infusion pumps in terms of residues in their external areas is crucial to promote the safety of all those who live with patients undergoing treatment. The presence of 5-FU residues in these areas could result in exposure risks for family members and caregivers, as well as for pets. Studies have shown worrying clinical conditions, including vomiting (with or without blood), convulsions, tremors, arrhythmias, and even death when pets accidentally ingest or come into topical contact with 5-FU [67,68,69]. Thus, the protection and safety of caregivers, family members and pets should not be neglected. Family members and caregivers of cancer patients undergoing treatment using 5-FU infusion pumps should be trained in waste management due to possible contact with the patient’s excreta. However, they must also be informed and trained verbally and in writing about the risks associated with exposure to cytotoxic drugs and how to act in the event of an incident with the infusion pump, such as a leak or spillage [61]. Still, none of the analyzed pumps in this study presented 5-FU detectable residues in the outer area (hard casing) and the infusion wire, indicating the absence of 5-FU spills and leaks in the area most exposed to the environment. These results also suggest that the pharmacy professionals who prepared the infusion pumps were able to do so without contaminating the casing and wire.

On the other hand, the areas of the ports connecting to the central catheter of all infusion pumps showed residues of 5-FU, except for pumps 1 and 5. This area of the infusion pumps is not usually exposed to the environment, and, therefore, family members and pets are less likely to be exposed to 5-FU. This result cannot be considered completely surprising since the flow of diluted 5-FU passes through this connection zone. These findings ultimately represent a positive control that suggests the suitability of the analytical method described herein. In addition, these findings put the focus, in terms of safety, on another group of individuals. The presence of 5-FU residues in the infusion port may raise concerns about the risk of exposure for nurses who disconnect the infusion pumps at the end of treatment. In this way, the exposure of these professionals and their surroundings increases the risk of occupational exposure to 5-FU. Studies have revealed the presence of mutagenicity and chromosomal alterations in the urine of nurses who do not use personal protective equipment (PPE) in oncology units [70,71,72]. There is also evidence of an impact on reproduction, causing abortions and fetal malformations [73,74]. PPE should be used when directly or indirectly handling or manipulating cytotoxic drugs, including 5-FU infusion pumps, because they provide a primary physical barrier to occupational exposure [61]. In addition to the use of PPE, the application of gauze pads during the disconnection of infusion pumps may serve to further reduce exposure to 5-FU residues, as is also recommended during the preparation and connection of syringes and needles at their junctions [75]. These procedures should be conducted in specifically designated, restricted-access areas, isolated from other patients. Furthermore, to minimize occupational and environmental contamination risks, infusion pumps must be disposed of in dedicated hazardous waste containers. Professionals who manipulate or handle cytotoxic drugs must receive training appropriate to their role. Disconnecting 5-FU infusion pumps in primary healthcare units, namely health centres, by nurses who do not have a specialization in oncology, should be discouraged. Even in hospital units specializing in oncology, the training of professionals is often portrayed as deficient, so in non-specialized units, the risks of inadequate training are certainly increased. A study by Kieffer et al. [76] showed that of the nine oncology units evaluated, 89% of healthcare professionals reported limited knowledge of the risks and handling of cytotoxic drugs, including waste for disposal, reinforcing the importance of intensive and recurrent training on cytotoxic drugs. In the package leaflet for the used infusion pumps, there was no mention of the possible leakage of residues during treatment or when removing the device. Despite the observed results, it should be emphasized that the methodology does not ensure confirmatory findings. Mass spectrometry analysis would be required for confirmation and to avoid false positives. The possibility of co-elution of multiple compounds with 5-FU cannot be disregarded.

This study can be considered a pilot study that demonstrates the applicability of the analytical method described for a fast analysis of the performance of infusion pumps in terms of residue retention, in the context of a screening method. A recent study described a confirmation method for the detection of 5-FU residues, which, although originally developed for surface contamination analysis, can be adapted as a confirmation method following a positive screening result using HPLC-DAD [77]. In addition, the results observed reinforce the importance of administrative controls and the correct use of PPE by nursing professionals when removing infusion pumps. These findings emphasize the significance of specialized training in cytotoxic drug administration. Providing data on potential cytotoxic drug leaks within device leaflets, directly from manufacturers, could also offer several advantages. It would demonstrate manufacturers’ commitment to the safety of patients’ families, the surrounding environment, and healthcare professionals. Confirmed leak-free performance could bolster patient confidence in outpatient device use. Conversely, identified leaks would prompt manufacturers to implement corrective measures or issue warnings.

In addition to the first description of an analytical method to assess the quality of infusion pumps in terms of residual leak retention, this study also presents a highly adaptable method for multiple contexts. Regardless of not being the most sensitive analytical chromatography equipment, HPLC-DAD systems are available in many institutions, including hospital units. The description of this method may encourage hospitals to perform their own tests. It is important to add that the method is quite simple in terms of sample treatment and extraction and efficient in terms of analysis, consisting of a chromatographic run with a duration of 10 min, which makes it feasible to analyze multiple samples in a short period of time.

## 5. Conclusions

In this study, an analytical methodology to accurately assess the actual occurrence of 5-FU leaks from infusion pumps was depicted. The developed methodology complies with all the analytical requirements recommended by the main organizations, and the method is easy and fast to implement. The application of this methodology will enable quality control of infusion pumps available on the market and, consequently, provide greater safety for family members and pets who live with the patient on a daily basis. Preliminary application of the methodology to a small number of samples did not detect any 5-FU residue in the hard casing and infusion wire. However, 5-FU was consistently detected in the catheter connection area. These findings reinforce the importance of adopting careful handling practices and the use of PPE by the nursing team responsible for removing infusion pumps to prevent repeated and potentially dangerous occupational exposure to 5-FU.

## Figures and Tables

**Table 1:** Back calculation of 5-FU calibration standards with deviations.

5-FU Concentration (µg/cm^2^)	Obtained 5-FU (µg/cm^2^) Concentration After Back-Calculation	Accordance (%)
0.150	0.154	102
0.375	0.364	97
0.750	0.783	104
1.125	1.138	101
1.500	1.427	95
2.250	2.289	102
3.000	2.995	100

**Table 2:** Repeatability (*n* = 9) and intermediate precision (*n* = 9) values developed for the validation of the HPLC-DAD analytical method for the detection and quantification of 5-FU.

Calibration Standards Concentration (µg/cm^2^)	Repeatability		Intermediate Precision	
	Mean ± Standard Deviation	RSD (%)	Mean ± Standard Deviation	RSD (%)
0.375	154,474.8 ± 4507.64	2.9	141,524.3 ± 17,912.83	12.7
1.125	331,210.9 ± 6055.42	1.8	308,320.7 ± 14,758.78	4.8
2.250	578,824.6 ± 18,221.2	3.2	520,997.1 ± 12,636.53	2.4

**Table 3:** Analytical data for accuracy of 5-FU determination.

5-FU Real Concentration (µg/cm^2^)	5-FU Experimentally Obtained Concentration (µg/cm^2^)	Relative Error (%)	Accuracy (%)
0.375	0.319	−14.9	85.1
1.125	1.094	−2.8	97.2
2.250	2.243	−0.3	99.7

**Table 4:** Analytical data for robustness evaluation regarding pH and flow rate parameters.

Parameter	Parameter Value	Peak Area	% of Peak Area in Relation to Optimized Method	Retention Time (min)	Difference of Retention Time in Relation to Optimized Method (min)
pH	2.4	321,416.33 ± 1532.37	NA	3.0	NA
	2.2	323,696.67 ± 512.60	100.7	3.0	0.0 min
Flow rate	0.8 mL/min	326,116.67 ± 677.66	NA	3.0	NA
	1.0 mL/min	226,990.33 ± 601.42	69.6	2.8	−0.2 min

NA, Not applicable.

**Table 5:** Analytical data for the stability of 5-FU after 3 and 5 d under three storage conditions (room temperature, 4 °C, and −20 °C), for three levels of concentration.

5-FU Concentration (µg/cm^2^)	Room Temperature		4 °C		−20 °C	
	Day 3 (%)	Day 5 (%)	Day 3 (%)	Day 5 (%)	Day 3 (%)	Day 5 (%)
0.150	109	77	94	71	96	61
0.750	105	96	97	89	95	85
3.000	90	86	84	83	83	84

**Table 6:** Results of the analysis of samples taken from three areas of real infusion pumps, analyzed by the HPLC-DAD method developed for the quantification of 5-FU *.

Infusion Pump ID	Concentration of 5-FU (µg/cm^2^) in the Analyzed Areas of the Infusion Pumps		
	Outside of the Pump (Hard Casing)	Infusion Wire	Catheter Connection Port
Infusion Pump 1	<LOD	<LOD	<LOD
Infusion Pump 2	<LOD	<LOD	0.39 *
Infusion Pump 3	<LOD	<LOD	0.22 *
Infusion Pump 4	<LOD	<LOD	0.62 *
Infusion Pump 5	<LOD	<LOD	<LOD
Infusion Pump 6	<LOD	<LOD	7.06 *
Infusion Pump 7	<LOD	<LOD	0.22 *
Infusion Pump 8	<LOD	<LOD	0.73 *
Infusion Pump 9	<LOD	<LOD	0.22 *
Infusion Pump 10	<LOD	<LOD	0.18 *

<LOD, below limit of detection (0.05 µg/cm^2^); * Results should be interpreted as screening data and must be subsequently confirmed by MS analysis.

**Table 7:** Limit of detection (LOD), limit of quantification (LOQ), analytical method, sample matrix, and main objective of different studies in which 5-fluorouracil (5-FU) was detected on gauze dressings.

Reference	LOD	LOQ	Analytical Method	Injection Volume	Study Main Goal
Present Study	0.050 µg/cm^2^	0.14 µg/cm^2^	HPLC-DAD	10 µL	Detection of 5-FU residues in external parts of infusion pumps
Viegas et al. [20,27]	0.010 µg/cm^2^	0.033 µg/cm^2^	HPLC-DAD	100 µL	Detection of 5-FU residues in room surfaces
Labrèche et al. [52]	0.040 ng/cm^2^	0.14 ng/cm^2^	LC-MS/MS	Not reported	Detection of 5-FU residues in room surfaces
Pinet et al. [53]	0.04 ng/cm^2^	0.099 ng/cm^2^	LC-MS/MS	Not reported	Detection of 5-FU residues in room surfaces
Rossignol et al. [54]	0.0013 ng/cm^2^	0.025 ng/cm^2^	LC-MS/MS	7 µL	Detection of 5-FU residues in room surfaces
Chauchat et al. [55]	0.040 ng/cm^2^	0.14 ng/cm^2^	LC-MS/MS	Not reported	Detection of 5-FU residues in room surfaces
Kåredal et al. [56]	0.0018 ng/cm^2^	0.0035 ng/cm^2^	LC-MS/MS	20 µL	Detection of 5-FU residues in room surfaces

## Data Availability

The data that support the findings of this study are available from the corresponding author upon reasonable request.
